# Supplementary figures and images for: Modeling the cis-regulatory modules of genes expressed in developmental stages of Drosophila melanogaster
Source: PeerJ. 2017 May 30;5:e3389. doi: 10.7717/peerj.3389 (PMC5452948; doi:10.7717/peerj.3389)

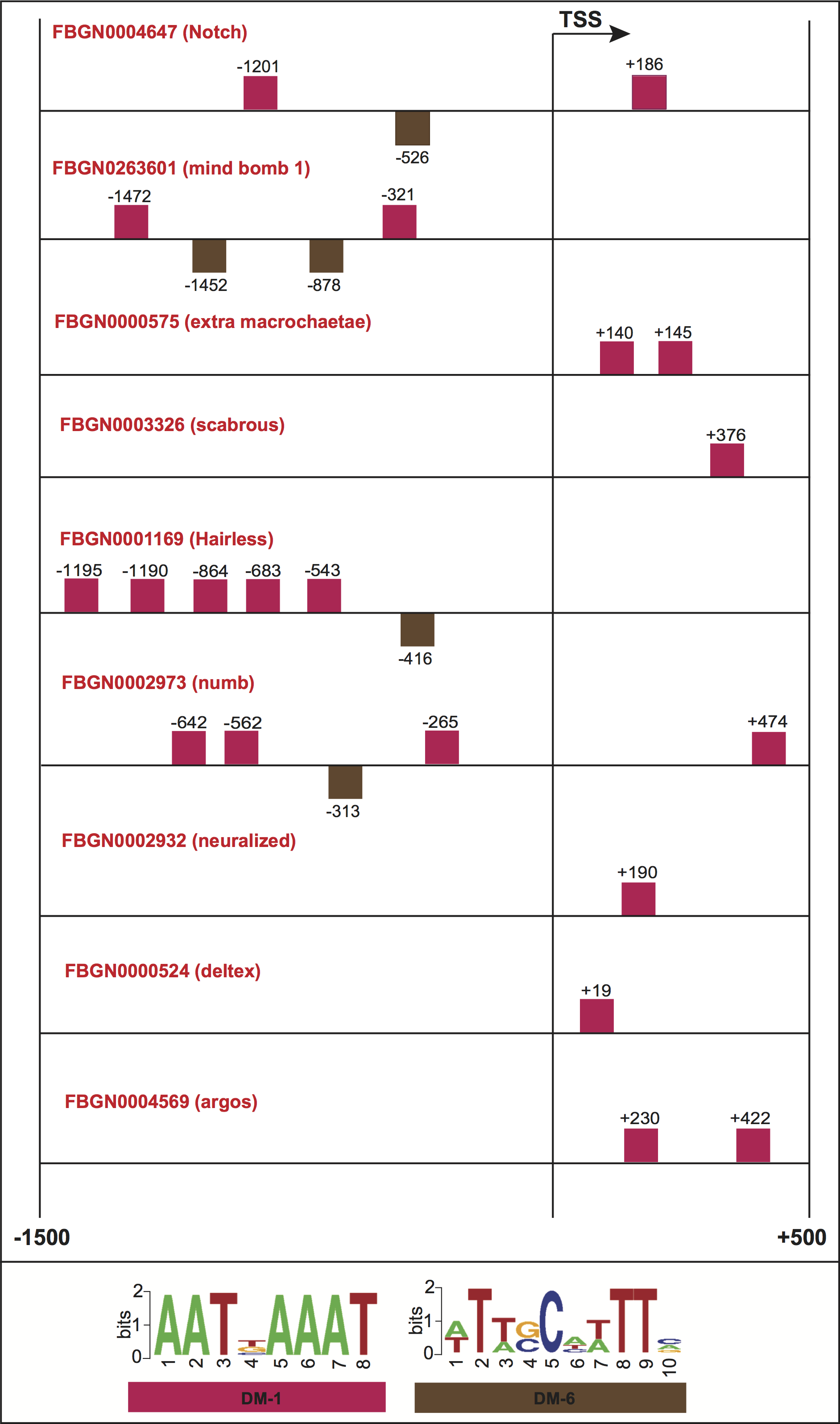

Supplement: Figure S1 — Squares above/below the horizontal line indicate the DNA strand where the motif is located. [file peerj-05-3389-s001.png]
