# Supplementary material for: Modeling the cis-regulatory modules of genes expressed in developmental stages of Drosophila melanogaster
Source: PeerJ. 2017 May 30;5:e3389. doi: 10.7717/peerj.3389 (PMC5452948; doi:10.7717/peerj.3389)
Supplement: Table S2 [file peerj-05-3389-s003.docx]

**Table S2. Number of genes in the different sets for each developmental stage.**

| **Developmental Stage** | **Seta** | **Setb** | **Setc** | **Setd** | **Sete** | **Setf** |
| --- | --- | --- | --- | --- | --- | --- |
| Embryo 0-2h | 90 | 4,490 | 88 (2) | 35 | 18 | 35 |
| Embryo 2-4h | 116 | 3,444 | 115 (1) | 46 | 23 | 46 |
| Embryo 4-6h | 129 | 2,986 | 128 (1) | 51 | 26 | 51 |
| Embryo 6-8h | 64 | 3,236 | 62 (2) | 25 | 12 | 25 |
| Embryo 8-10h | 45 | 2,891 | 44 (1) | 18 | 9 | 17 |
| Embryo 10-12h | 206 | 2,726 | 203 (3) | 81 | 41 | 81 |
| Embryo 12-14h | 84 | 2,416 | 83 (1) | 33 | 17 | 33 |
| Embryo 14-16h | 268 | 2,684 | 262 (6) | 105 | 52 | 105 |
| Embryo 16-18h | 155 | 2,320 | 150 (5) | 60 | 30 | 60 |
| Embryo 18-20h | 116 | 2,362 | 112 (4) | 45 | 22 | 45 |
| Embryo 20-22h | 173 | 2,375 | 171 (2) | 68 | 34 | 69 |
| Embryo 22-24h | 229 | 2,204 | 226 (3) | 90 | 45 | 91 |
| L1 stage larvae | 237 | 2,406 | 234 (3) | 94 | 47 | 93 |
| L2 stage larvae | 204 | 2,358 | 202 (2) | 81 | 40 | 81 |
| L3 stage larvae | 204 | 761 | 200 (4) | 80 | 40 | 80 |
| White prepupae | 280 | 1,265 | 271 (9) | 108 | 54 | 109 |
| White prepupae + 12h | 268 | 995 | 259 (9) | 104 | 52 | 103 |
| White prepupae + 24h | 284 | 1,174 | 274 (10) | 110 | 55 | 109 |
| Pupae | 338 | 532 | 324 (14) | 130 | 65 | 129 |
| Adult male eclosion + 1 day | 355 | 909 | 336 (19) | 134 | 67 | 135 |
| Adult male eclosion + 5 days | 336 | 700 | 317 (19) | 127 | 63 | 127 |
| Adult male eclosion + 30 days | 353 | 932 | 336 (17) | 134 | 67 | 135 |
| Adult female eclosion + 1 day | 200 | 2,525 | 197 (3) | 79 | 39 | 79 |
| Adult female eclosion + 5 days | 188 | 2,780 | 186 (2) | 74 | 37 | 75 |
| Adult female eclosion + 30 days | 222 | 2,576 | 219 (3) | 88 | 44 | 87 |
| **Seta:** initial set of expressed genes  **Setb:** control set  **Setc:** filtered genes (removed genes)  **Setd:** motif-prediction set  **Sete:** feature-computation set  **Setf:** model-construction set | | | | | | |
