# Supplementary material for: Modeling the cis-regulatory modules of genes expressed in developmental stages of Drosophila melanogaster
Source: PeerJ. 2017 May 30;5:e3389. doi: 10.7717/peerj.3389 (PMC5452948; doi:10.7717/peerj.3389)
Supplement: Table S4 [file peerj-05-3389-s005.docx]

**Table S4. Number of *de novo*, non-redundant, overrepresented motifs and motifs included in the informative features of each model.**

| **Developmental Stage** | **Ma** | **Mb** | **Mc** | **Md** |
| --- | --- | --- | --- | --- |
| Embryo 0-2h | 176 | 57 | 29 | 10 |
| Embryo 2-4h | 177 | 69 | 28 | 5 |
| Embryo 4-6h | 202 | 59 | 21 | 9 |
| Embryo 6-8h | 185 | 70 | 34 | 6 |
| Embryo 10-12h | 210 | 48 | 6 | 6 |
| Embryo 12-14h | 175 | 47 | 18 | 6 |
| Embryo 14-16h | 201 | 51 | 5 | 4 |
| Embryo 16-18h | 194 | 48 | 12 | 9 |
| Embryo 18-20h | 181 | 49 | 15 | 6 |
| Embryo 20-22h | 185 | 42 | 8 | 7 |
| Embryo 22-24h | 181 | 47 | 13 | 10 |
| L1 stage larvae | 200 | 51 | 4 | 3 |
| L2 stage larvae | 191 | 56 | 9 | 6 |
| L3 stage larvae | 199 | 45 | 7 | 3 |
| White prepupae | 186 | 43 | 6 | 2 |
| White prepupae + 12h | 186 | 40 | 4 | 3 |
| White prepupae + 24h | 198 | 36 | 6 | 4 |
| Pupae | 178 | 41 | 8 | 4 |
| Adult male eclosion + 1 day | 192 | 37 | 6 | 5 |
| Adult male eclosion + 5 days | 200 | 40 | 6 | 5 |
| Adult male eclosion + 30 days | 203 | 44 | 6 | 5 |
| Adult female eclosion + 1 day | 191 | 56 | 15 | 10 |
| Adult female eclosion + 5 days | 189 | 64 | 21 | 9 |
| Adult female eclosion + 30 days | 202 | 41 | 9 | 8 |
| **Ma:** *de novo* motifs  **Mb:** non-redundant motifs  **Mc:** overrepresented motifs  **Md:** motifs included in the informative features | | | | |
