# Supplementary material for: Modeling the cis-regulatory modules of genes expressed in developmental stages of Drosophila melanogaster
Source: PeerJ. 2017 May 30;5:e3389. doi: 10.7717/peerj.3389 (PMC5452948; doi:10.7717/peerj.3389)
Supplement: Table S8 — Number of genes with each annotation (Count), uncorrected and corrected (Bonferroni) p-values are shown. [file peerj-05-3389-s009.docx]

**Table S8.** **Gene ontology terms of the genes involved in Notch signaling system.** Number of genes with each annotation (Count), uncorrected and corrected (Bonferroni) *p*-values are shown.

| **Gene Ontology Term** | **Count** | ***P*-Value** | **Bonferroni** |
| --- | --- | --- | --- |
| Notch signaling pathway | 7 | 1.8E-12 | 3.4E-10 |
| lateral inhibition | 7 | 1.3E-9 | 2.4E-7 |
| positive regulation of Notch signaling pathway | 4 | 2.9E-6 | 5.5E-4 |
| regulation of Notch signaling pathway | 4 | 2.9E-6 | 5.5E-4 |
| Developmental protein | 6 | 6.9E-6 | 2.8E-4 |
| imaginal disc-derived wing morphogenesis | 5 | 9.7E-6 | 1.9E-3 |
| R8 cell fate commitment | 3 | 9.7E-6 | 1.9E-3 |
| peripheral nervous system development | 4 | 2.3E-5 | 4.5E-3 |
| Phosphoprotein | 6 | 5.6E-5 | 2.2E-3 |
| protein binding | 6 | 7.6E-5 | 1.4E-3 |
